# 4‐PBA inhibits hypoxia‐induced lipolysis in rat adipose tissue and lipid accumulation in the liver through regulating ER stress

**DOI:** 10.1002/fsn3.3156

**Published:** 2023-02-09

**Authors:** Yanlei Xiong, Yueming Wang, Yanlian Xiong, Lianghong Teng

**Affiliations:** ^1^ Department of Pathology Xuanwu Hospital, Capital Medical University Beijing China; ^2^ Department of Pathophysiology Institute of Basic Medical Sciences, Chinese Academy of Medical Sciences (CAMS); School of Basic Medicine, Peking Union Medical College (PUMC) Beijing China; ^3^ Department of Anatomy School of Basic Medicine, Binzhou Medical University Yantai China

**Keywords:** endoplasmic reticulum stress, hypoxia, lipolysis, liver dysfunction, white adipose tissue

## Abstract

High‐altitude hypoxia may disturb the metabolic modulation and function of both adipose tissue and liver. The endoplasmic reticulum (ER) is a crucial organelle in lipid metabolism and ER stress is closely correlated with lipid metabolism dysfunction. The aim of this study is to elucidate whether the inhibition of ER stress could alleviate hypoxia‐induced white adipose tissue (WAT) lipolysis and liver lipid accumulation‐mediated hepatic injury. A rat model of high‐altitude hypoxia (5500 m) was established using hypobaric chamber. The response of ER stress and lipolysis‐related pathways were analyzed in WAT under hypoxia exposure with or without 4‐phenylbutyric acid (PBA) treatment. Liver lipid accumulation, liver injury, and apoptosis were evaluated. Hypoxia evoked significant ER stress in WAT, evidenced by increased GRP78, CHOP, and phosphorylation of IRE1α, PERK. Moreover, Lipolysis in perirenal WAT significantly increased under hypoxia, accompanied with increased phosphorylation of hormone‐sensitive lipase (HSL) and perilipin. Treatment with 4‐PBA, inhibitor of ER stress, effectively attenuated hypoxia‐induced lipolysis via cAMP‐PKA‐HSL/perilipin pathway. In addition, 4‐PBA treatment significantly inhibited the increase in fatty acid transporters (CD36, FABP1, FABP4) and ameliorated liver FFA accumulation. 4‐PBA treatment significantly attenuated liver injury and apoptosis, which is likely resulting from decreased liver lipid accumulation. Our results highlight the importance of ER stress in hypoxia‐induced WAT lipolysis and liver lipid accumulation.

## Graphical abstract

Enhanced ER stress mediated WAT lipolysis was observed in a rat model of high‐altitude hypoxia, which contribute to hepatic dysfunction and apoptosis through excess release of FFA. Our findings highlight the vital role of 4‐PBA in WAT lipolysis and liver dysfunction via regulating ER stress, which may provide novel insights into systemic metabolic disturbances in high‐altitude area.

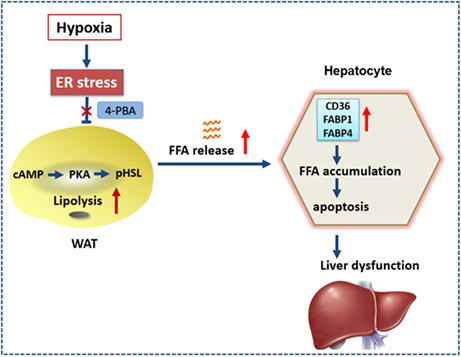

**Abbreviations**
CD36: cluster of differentiation
CHOP: CCAAT/enhancer‐binding protein homologous protein
ER: endoplasmic reticulum
FABP1: fatty acid binding protein 1
FABP4: fatty acid binding protein 4
FFA: Free fatty acids
GRP78: glucose‐regulated protein 78
HSL: Hormone‐sensitive lipase
IRE1α: inositol‐requiring enzyme 1α
PERK: protein kinase RNA (PKR) ‐like ER kinase
PKA: cAMP‐dependent protein kinase A
TG: Triacylglycerol
UPR: unfolded protein response
WAT: White adipose tissue

## 1 INTRODUCTION

Ascent to high altitude is associated with multi physiological and metabolic responses to counter with the stress of hypobaric hypoxia. White adipose tissue (WAT) is the largest reservoir of energy reserves, which stores energy in the form of triglyceride in lipid droplets. WAT plays an essential role in maintaining the whole‐body lipid metabolism homeostasis and accumulated evidence has demonstrated the functional association between adipose tissue and liver (Natarajan et al., 2017; Sun et al., 2012). In our previous work, hypobaric hypoxia was proved to accelerate lipolysis and suppress lipogenesis of WAT (Xiong et al., 2014). Under normal conditions, the lipid metabolism is a dynamic equilibrium process between different organs. However, under hypoxia environment, the activation of lipolysis promotes excessive free fatty acids (FFA) release, which is taken up by the liver, contributing to ectopic lipid accumulation and pathogenesis of liver (Lefere et al., 2016). Adipose tissue dysfunction could lead to increased delivery of FFA and glycerol to the liver which drives hepatic gluconeogenesis and facilitates the accumulation of lipids and insulin signaling inhibiting lipid intermediates (Bosy‐Westphal et al., 2019). Herein, hypoxia caused lipid metabolism disorder of WAT may further influence liver function, leading to the maladaptation to high‐altitude environment and increasing the incidence of acute mountain sickness (AMS).

The endoplasmic reticulum (ER) is an organelle that functions to synthesize, fold, and transport proteins. It is also the site of triglyceride synthesis and nascent lipid droplet formation (Nettebrock & Bohnert, 2019). The sensing, metabolizing, and signaling mechanisms for lipid metabolism exist within or on the ER membrane domain (Balla et al., 2020). Dysregulation of ER homeostasis led to accumulation of misfolded proteins in the ER lumen and evoke ER stress (Henne, 2019). To reduce ER stress, the unfolded protein response (UPR) signal pathways are activated. Recently, accumulated evidence suggested that ER homeostasis and UPR activation play an important homeostatic role in lipid metabolism (Basseri & Austin, 2012; Mohan et al., 2019). As reported by Deng et al., ER stress could induce lipolysis by activating cAMP/PKA and ERK1/2 pathways (Deng et al., 2012). Previous study also found that burned patients displayed significant ER stress within adipose tissue and ER stress could augment lipolysis in cultured human adipocytes (Bogdanovic et al., 2015).

The disulfide bond formation during protein synthesis is independent of oxygen, however, the post‐translational protein folding and isomerization process is oxygen‐dependent (Koritzinsky et al., 2013). Herein, hypoxia exposure could induce extensive protein modification in the ER and result in the accumulation of misfolded/unfolded proteins, which activate UPR and evoke ER stress (Chipurupalli et al., 2019; Maekawa & Inagi, 2017). We decided to test the hypothesis that ER stress may modulate hypoxia‐induced WAT metabolic derangement and liver dysfunction based on the following evidence: (1) ER is one of the major sites of lipid metabolism. (2) lipid metabolism and function are sensitive to oxygen concentration. (3) Hypoxia could induce ER stress due to the accumulation of misfolded proteins (Xu et al., 2015; Yang et al., 2014). (4) ER stress is closely correlated with lipid metabolism dysfunction (Mohan et al., 2019). (5) lipid metabolism in WAT plays a critical role in the progression of liver dysfunction (Dong et al., 2020).

To address this issue, we investigated the effects of ER stress in hypoxia‐induced lipolysis using chemical chaperone 4‐PBA, antagonist of ER stress. The main objective of this study was to clarify the role of ER stress which regulates WAT lipolysis and liver lipid accumulation under continuous high‐altitude hypoxia exposure. An understanding of the interplay between tissues and these proposed mechanisms may provide novel therapeutic strategies for the treatment of the whole‐body metabolism dysfunction at high altitude.

## 2 MATERIALS AND METHODS

### 2.1 Animals care

Adult male Sprague–Dawley rats (280–330 g) were purchased from Weitong Lihua Laboratory Animal Limited Company. The rats were housed at room temperature (22°C–25°C) and in a 12–12 h light–dark cycle with free access to food and water and adapted to the condition above for 1 week before experiment. All experiments were conducted in strict accordance with the laboratory animal care guidelines published by the US National Institutes of Health (NIH publication no. 85–23, revised 1996). All protocols concerning animal use were approved by the Institutional Animal Care and Use Committee of Institute of Basic Medical Sciences, Peking Union Medical College and Capital Medical University.

### 2.2 Hypoxic challenge

Hypoxia group rats were placed in a hypobaric chamber (Guizhou Fenglei Air Ordnance Co., Ltd.) and subjected to hypoxia mimicking an altitude of 5500 m for 10 days. The chamber was opened daily for 30 min to clean and replenish food and water and room temperature was kept at 20°C–22°C. We monitored the body weights of rats every day. 4‐PBA (P21005) was commercially purchased (Sigma‐Aldrich). Rats were randomly divided into four groups: (1) Control group, (2) Hypoxia group, (3) Control + 4‐PBA (30 mg/kg /day), and (4) Hypoxia + 4‐PBA (30 mg/kg/day). The dose of 4‐PBA was set based on previous reports (Luo et al., 2015; You et al., 2019; Zeng et al., 2017). All the rats were sacrificed by decapitation and serum was obtained by centrifugation and stored at −80°C. The perirenal fat pads were collected and weighed immediately, frozen in liquid nitrogen, and stored at −80°C.

### 2.3 Histology staining

WAT and liver tissue were fixed in 4% paraformaldehyde overnight, followed by embedment in paraffin and longitudinal slicing, with 4‐μm‐thick sections obtained for hematoxylin‐eosin (HE) staining. The stained slides were examined by microscopy for histomorphological analyses. A commercial terminal deoxynucleotidyl transferase‐mediated dUTP nick‐end labeling (TUNEL) kit (Roche) was employed to assess the degree of hepatic cell apoptosis. Histological alterations were assessed in randomly selected histological fields at ×400 magnification and apoptosis index (AI) was calculated.

### 2.4 Western blotting and densitometry analyses

Homogenized rat WAT was lysed in 200 μl RIPA lysis buffer (Beyotime, P0013B) with 1% phenylmethyl sulfurylfluoride and 4% complete protease inhibitor cocktail mix (Roche). Extracts were centrifuged at 14,000 g for 15 min at 4°C. Eighty micrograms of total protein was used for sodium dodecyl sulfate‐polyacrylamide gel electrophoresis, followed by transferring blotting to nitrocellulose membrane (Millipore Corp., Billerica). Membranes were then blocked with 5% non‐fat‐dried milk in PBS for 1 h with gentle shaking. Membranes were incubated first with primary antibodies (dilution: 1:1000) overnight at 4°C, in 1% BSA in PBS overnight at 4°C with shaking. The following primary antibodies were purchased from Cell Signaling Technology: anti‐p‐HSL (#4139), anti‐HSL (#18381), anti‐pPKA, anti‐perilipin, anti‐Phospho‐PKA Substrate (RRXS*/T*) (#9624), anti‐GRP78 (#3183 S), anti‐CHOP (#2895P), anti‐protein kinase‐like eIF2α kinase (PERK) (#3192 S), and their phosphorylated species. anti‐ATGL antibody (ab109251), anti‐CGI58 antibody (ab111984), and anti‐β‐actin antibody (ab6276) were purchased from Abcam. Then, membranes were washed and incubated with secondary antibodies for 2 h at room temperature. Finally, the samples were visualized by enhanced chemiluminescence using Tanon‐410 automatic gel imaging system (Shanghai Tianneng Corporation). After scanning, band density was analyzed using Image J 1.33 software (National Institutes of Health).

### 2.5 Reverse‐transcription PCR and quantitative real‐time PCR

Total RNA was prepared from frozen liver tissues with TRIZOL (Invitrogen) reagent and the cDNA was synthesized using TransScript TM First‐Strand cDNA Synthesis Super‐Mix (TransGen Biotech, AT301). The program was run on a S1000 Thermal Cycler. Quantitative real‐time PCR was performed using the SYBR®Pre‐mix Ex TaqTMkit (Takara, RR420A) and analyzed in a step‐one plus RT‐PCR system (life science, Applied Biosystems). The primer sequences are listed in Table 1.

**TABLE 1:** Primer sequences (GenBank/NCBI) used in real‐time PCR

Primer ID	Primer sequence 5′–3′	Accession No.	Product size (bp)
CD36	Fed: TCCTCGGATGGCTAGCTGATT	NC_051339.1	150
	Rev: TGCTTTCTATGTGGCCTGGTT		
FABP1	Fed: CTTCTCCGGCAAGTACCAAGT	NM_012556.2	162
	Rev: CATGCACGATTTCTGACACCC		
FABP4	Fed: GTAGAAGGGGACTTGGTCGTC	NM_053365.2	234
	Rev: GCCTTTCATGACACATTCCAC		
β‐Actin	Fed: CGTTGACATCCGTAAAGACC	NM_031144.3	260
	Rev: GCTAGGAGCCAGGGCAGTA		

### 2.6 Serum measurements

Serum levels of non‐esterified fatty acid (NEFA) and glycerol were measured using NEFA kit (A042, Jiancheng Biotechnology) and Glycerol Assay kit (F005‐1, Jiancheng Biotechnology), respectively. These assays were performed according to manufacturer's instructions. Serum levels of triglyceride (TG), total cholesterol (TC), high‐density lipoprotein cholesterol (HDL‐C), and low‐density lipoprotein cholesterol (LDL‐C) were measured by an automatic biochemical analyzer (Chemray 240, Rayto Life and Analytical Sciences).

Serum alanine (ALT), aspartate aminotransferase (AST), and alkaline phosphatase (ALP) microplate test kits were obtained from Nanjing Jiancheng Bioengineering Institute. These assays were performed as previously described (Wang et al., 2020). Briefly, ALT, AST, and ALP activities were evaluated at 37°C for 15 min by assessing for a decrease in absorbance at a wavelength of 510 nm, with Chemi Lab ALT, AST, and ALP assay kits, respectively.

### 2.7 Statistical analysis

The data are presented as mean ± standard error (SE). For Western blot, protein levels were normalized to β‐actin. Statistical significance is determined by one‐way Analysis of variance (ANOVA) or nonparametric for more than three groups. *p*‐Value < .05 was considered statistically significant (SPSS 18.0 software).

## 3 RESULTS

### 3.1 Hypoxia exposure induces endoplasmic reticulum stress in WAT

**FIGURE 1:**
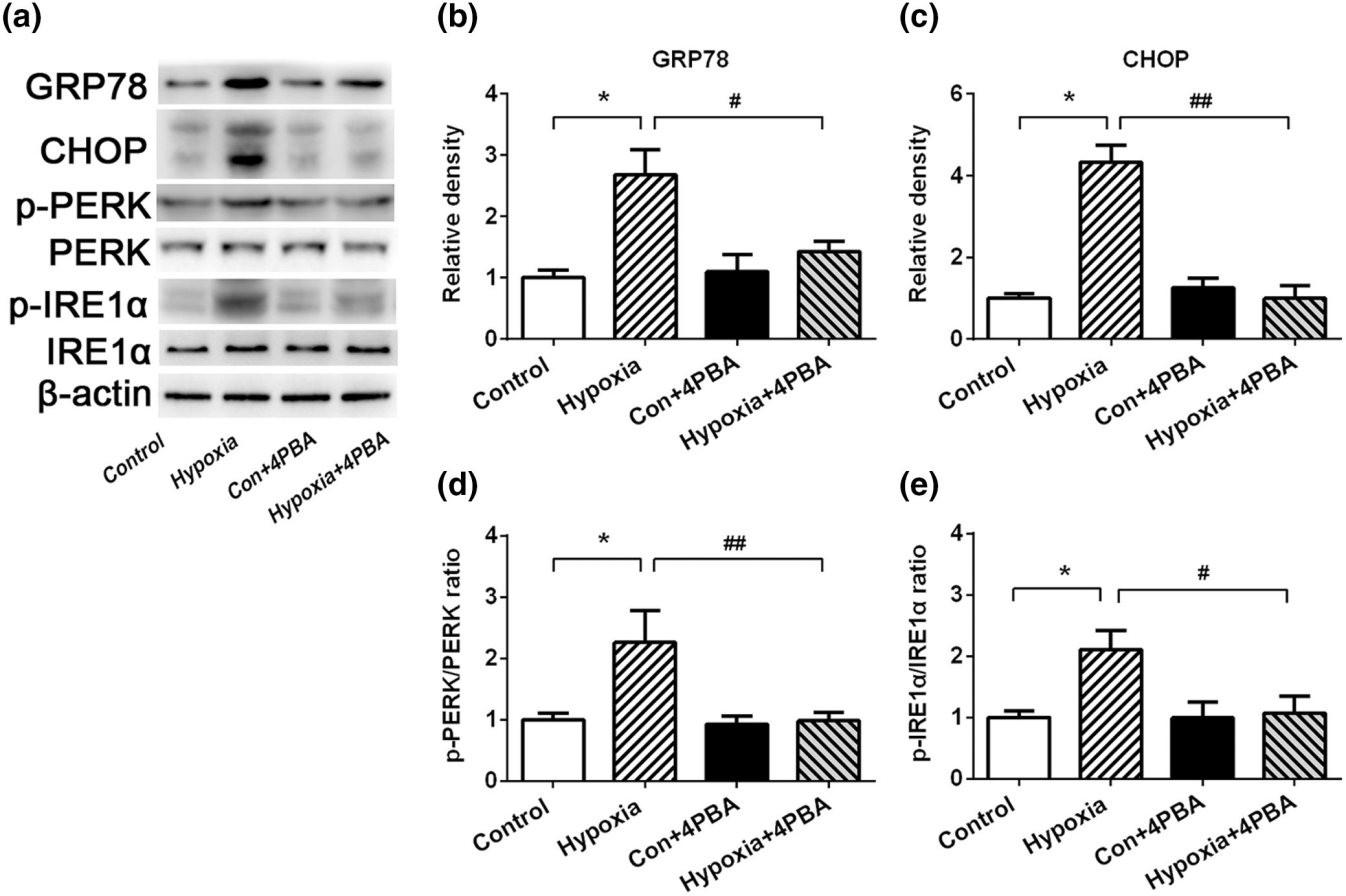
Hypoxia exposure induces endoplasmic reticulum stress in the WAT. The expression levels of ER stress‐related genes in WAT are shown. (a) GRP78, CHOP, p‐PERK, PERK, p‐IRE1α, and IRE1α protein expression levels; (b) Relative GRP78 protein expression levels; (c) relative CHOP protein expression levels; (d) p‐PERK/PERK ratio; (e) p‐IRE1α/IRE1α ratio. Data are shown as the mean ± SE of at least two independent western blots, *p < .05, **p < .01, and ***p < .001 (control group vs. hypoxia group, *n* = 6/group). ^#^ *p* < .05, ^##^ *p* < .01 (hypoxia group vs. hypoxia + 4‐PBA group, *n* = 6/group)

To investigate the role of ER stress in WAT under hypoxia treatment, we first examined the expression of ER stress markers, namely GRP78 and CHOP (Figure 1a). Under ER stress conditions, increased GRP78 is dissociated from unfolded proteins and activates ER stress receptors triggering the UPR. As shown in Figure 1b,c, hypoxia exposure significantly increased levels of GRP78 and CHOP. Continuous hypoxia treatment also activated ER stress‐related pathways in rat adipose tissue, evidenced by enhanced p‐PERK/PERK ratio (Figure 1d) and p‐IRE1α/IRE1α ratio (Figure 1e). 4‐PBA treatment significantly attenuated hypoxia‐induced ER stress, evidenced by decreased GRP78, CHOP, p‐PERK/PERK ratio, and p‐IRE1α/IRE1α ratio in 4‐PBA + hypoxia group as compared with hypoxia group.

#### 3.1.1 PBA treatment attenuates enhanced lipolysis in WAT induced by hypoxia

**FIGURE 2:**
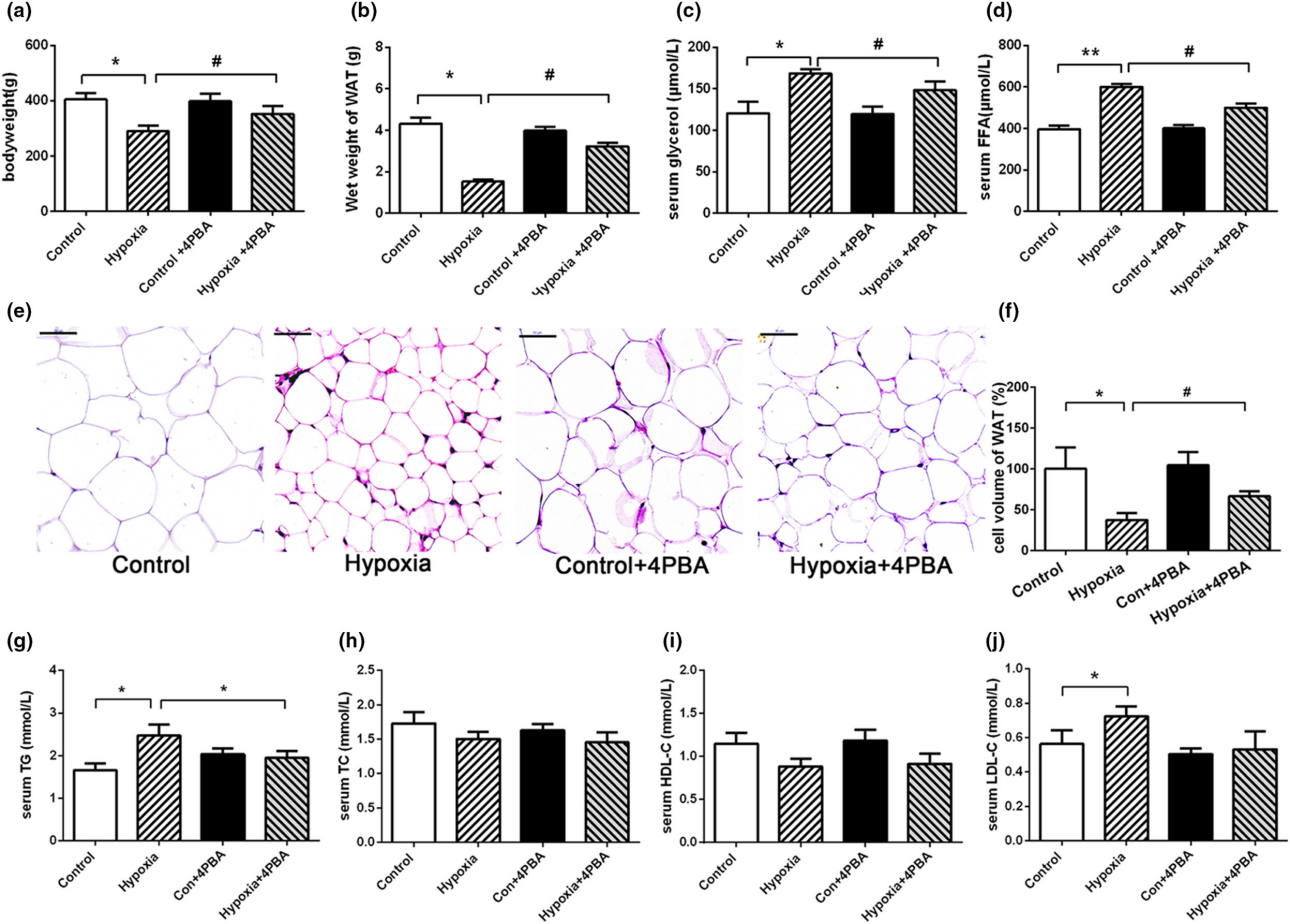
4‐PBA treatment attenuates enhanced lipolysis in white adipose tissue under hypoxia. 4‐PBA treatment (30 μg/kg body weight by intra‐peritoneal injection) significantly attenuates hypoxia‐induced body weight (a) and WAT loss (b); (c) serum levels of glycerol; (d) serum levels of FFA; (e) representative images of HE‐stained sections of WAT (magnification, 400×); (f) changes in the adipocytes volume in WAT. (g) Serum levels of TG (h); (i) serum levels of TG; (j) serum levels of HDL‐C; (J) serum levels of LDL‐C. Data are shown as mean ± SE, *p < .05, **p < .01, (control group vs. hypoxia group, *n* = 6/group). #*p* < .05, ##*p* < .01 (hypoxia group vs. hypoxia + 4‐PBA group, *n* = 6/group)

Compared with control group, exposure to hypoxia equivalent to an altitude of 5500 m for 10 days significantly reduced the body weight of rat and wet weight of perirenal fat (Figure 2a,b). Both serum levels of glycerol and FFA significantly increased in hypoxia group rats, indicating enhanced lipolysis under hypoxia exposure (Figure 2c,d). In support of these findings, histological analysis of WAT showed that continuous hypoxia significantly reduced the volume of adipocytes compared with that in control group rats (Figure 2e,f). Hypoxia exposure led to increased serum levels of triglycerides (TG), low‐density lipoprotein cholesterol (LDL‐C), while the levels of total cholesterol (TC) level and high‐density lipoprotein cholesterol (HDL‐C) did not change significantly (Figure 2g–j).

To investigate the effect of inhibition of ER stress on WAT lipolysis under hypoxia, we first evaluated the body weight and wet weight of perirenal fat in hypoxia rats with or without 4‐PBA treatment. 4‐PBA significantly attenuated the reduction of body weight and wet weight of perirenal fat after 10 days exposure to hypoxia (Figure 2a,b). In addition, inhibition of ER stress via 4‐PBA was associated with a significant reduction of lipolysis, evidenced by a significant reduction in serum glycerol and FFA levels (Figure 2c,d). Moreover, 4‐PBA treatment significantly attenuated hypoxia caused reduction of adipocyte volume (Figure 2f). 4‐PBA treatment effectively attenuated hypoxia‐induced increased levels of TG (Figure 2j).

### 3.2 ER stress inhibition ameliorate hypoxia‐induced WAT lipolysis via cAMP/PKA pathway

**FIGURE 3:**
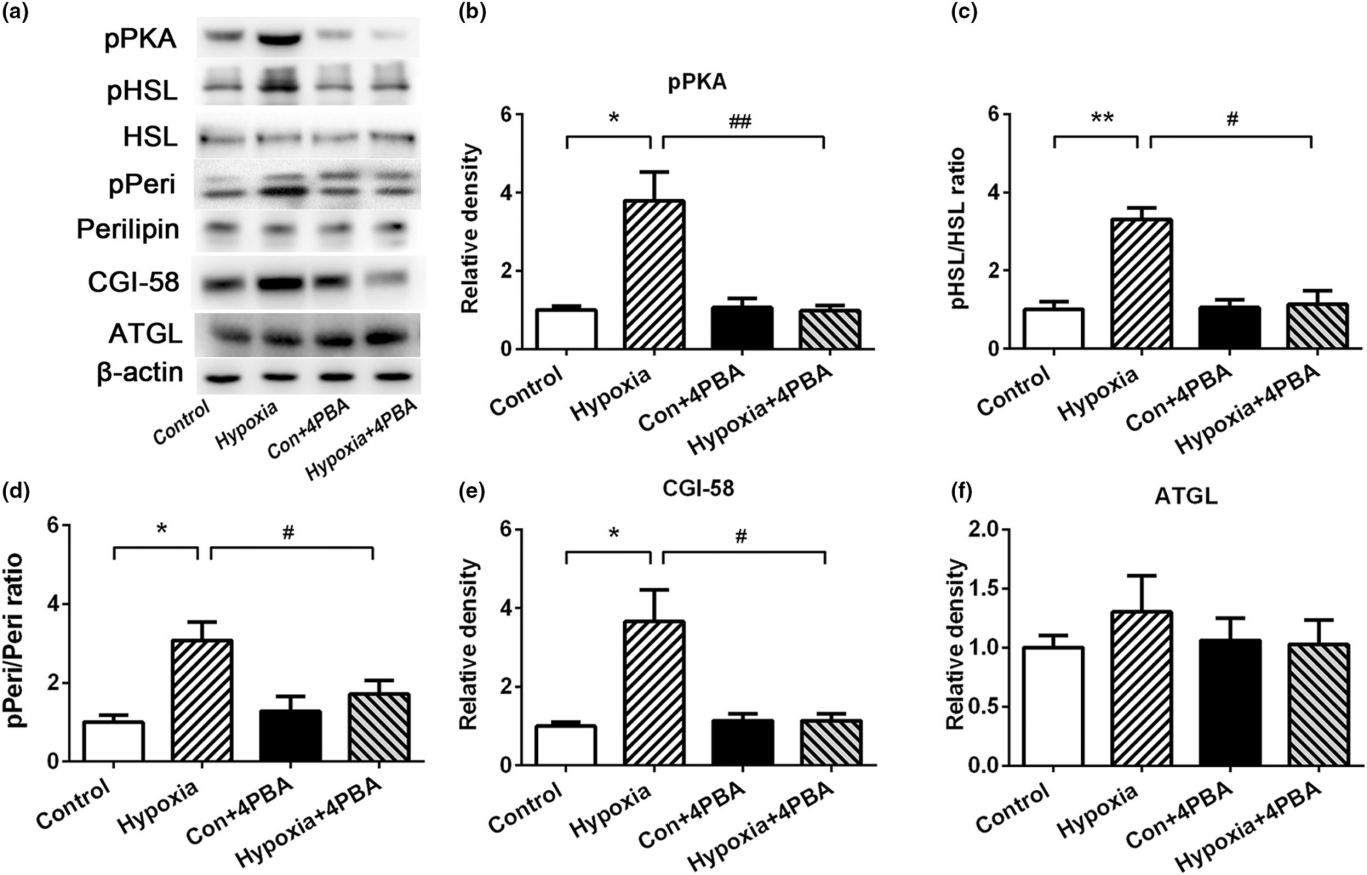
ER stress inhibition ameliorates WAT lipolysis in hypoxia rats via cAMP/PKA pathway. 4‐PBA treatment significantly downregulated expression levels of WAT lipolysis‐related genes induced by hypoxia. (a) pPKA, p‐HSL, HSL, p‐Peri, Perilipin, CGI‐58, and ATGL protein expression levels; (b) Relative pPKA protein expression levels; (c) p‐HSL/HSL ratio; (d) p‐Peri/Peri ratio; (e) Relative CGI‐58 protein expression levels; (f) Relative ATGL protein expression levels. Data are shown as the mean ± SE, *p < .05, **p < .01, and ***p < .001(control group vs. hypoxia group, *n* = 6/group). #*p* < .05, ##*p* < .01 (hypoxia group vs. hypoxia + 4‐PBA group, *n* = 6/group)

Endoplasmic reticulum stress has been suggested to trigger lipolysis in adipocytes. The lipolysis process is closely correlated with the production of cAMP and activation of cAMP‐dependent protein kinase A (PKA). In our study, hypoxia challenge significantly increased pPKA production (Figure 3a,b), which phosphorylates HSL and perilipin (Miyoshi et al., 2006; Sztalryd et al., 2003). The p‐HSL/HSL ratio (Figure 3c) and p‐Perilipin/Perilipin (Figure 3d) significantly increased in the hypoxic group, which were attenuated by 4‐PBA treatment. Although the abundance of ATGL remained unchanged in the WAT of the hypoxia rats, the level of CGI‐58 significantly increased in the hypoxia rats compared with the control rats (Figure 3e,f). Taken together, these data indicated that the inhibition of ER stress was shown to alleviate hypoxia‐induced lipolysis mainly by blocking the activation of cAMP‐PKA‐pHSL/Perilipin pathway.

#### 3.2.1 PBA treatment ameliorated hypoxia‐induced liver lipid transport and accumulation

**FIGURE 4:**
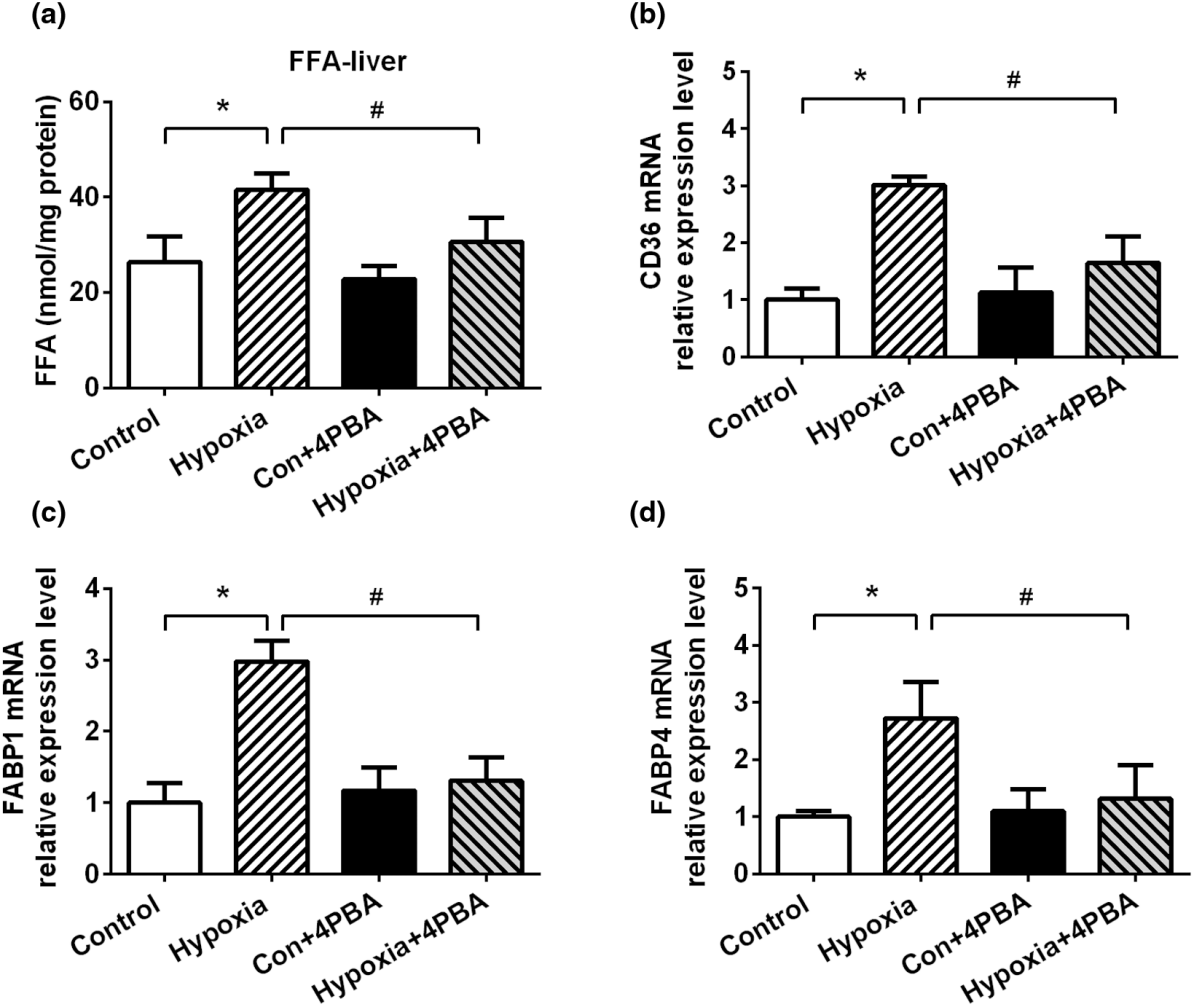
4‐PBA treatment ameliorates hypoxia‐induced liver lipid accumulation in the liver. (a) 4‐PBA treatment significantly attenuates hypoxia‐induced FFA accumulation in the liver. Relative mRNA levels of (b) CD36 (c) FABP1 and (d) FABP4. Data are shown as the mean ± SE, *p < .05, **p < .01, and ***p < .001(control group vs. hypoxia group, *n* = 6/group). #*p* < .05, ##*p* < .01 (hypoxia group vs. hypoxia + 4‐PBA group, *n* = 6/group)

Under continuous hypoxia exposure, increased delivery of free fatty acids (FFA) caused by enhanced lipolysis in WAT may contribute to the lipid accumulation in the liver. As shown in Figure 4a, levels of FFA content significantly increased in hypoxia group rat liver, which was attenuated by 4‐PBA treatment. Lipid uptake in the liver was regulated by many transporters, including cluster of differentiation (CD36), fatty acid binding protein 1(FABP1), and FABP4. mRNA levels of CD36, FABP1, and FABP4 that regulate the entry of fatty acids into hepatocyte, are generally upregulated to cope with increased circulation FFAs (Figure 4b–d).

#### 3.2.2 PBA treatment ameliorated hypoxia‐induced live hepatic injury and apoptosis

**FIGURE 5:**
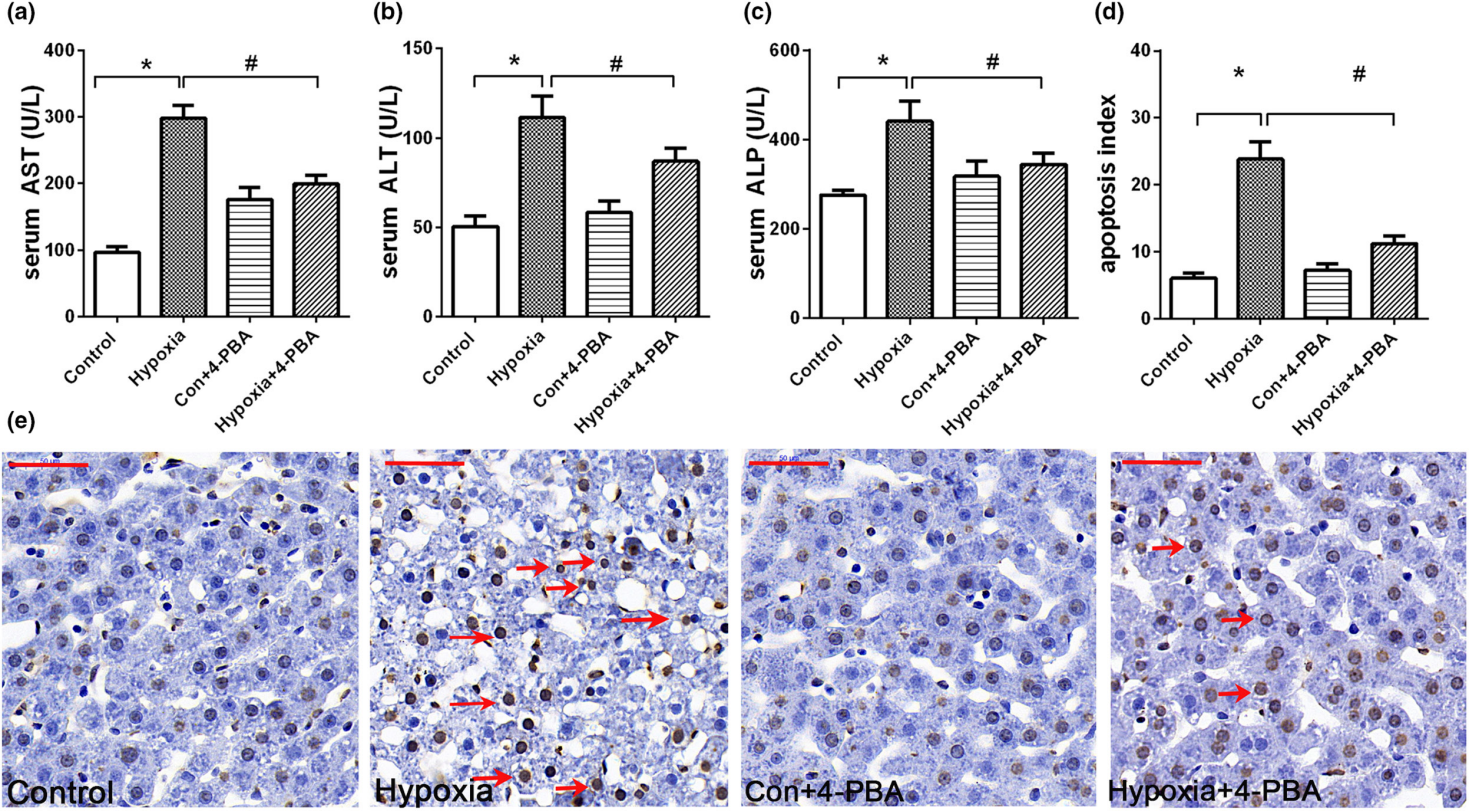
4‐PBA ameliorates hypoxia‐induced hepatic injury and apoptosis. (a) Serum levels of AST in rats exposed to hypoxic (*n* = 6) or normoxic (*n* = 6) conditions. (b) Serum levels of ALT; (c) serum levels of ALP; (d)apoptosis index of four group rats; (e) representative images of TUNEL‐stained sections of liver (magnification, 400×). Data are shown as mean ± SE, *p < .05, **p < .01, (control group vs. hypoxia group). #*p* < .05, ##*p* < .01 (hypoxia group vs. hypoxia + 4‐PBA group)

Hypoxia‐induced liver lipid accumulation may further trigger the pathogenesis of liver injury, serum levels of liver enzyme were tested to confirm our speculation. As shown in Figure 5a–c, the hypoxia group rat exhibited a marked increase in the levels of AST, ALT, and ALP (*p*<.05), indicating potential liver injury. However, the hypoxia + 4‐PBA group significantly decreased the levels of AST and ALT (*p*<.05) when compared with hypoxia group, indicating that 4‐PBA inhibits hypoxia‐induced hepatocellular injury.

The apoptosis status of rat liver exposed to hypoxia was evaluated with a TUNEL assay. As shown in Figure 5d,e, the percentage of apoptotic cells was significantly increased in hypoxia group as compared with control group, which was effectively attenuated by 4‐PBA treatment.

## 4 DISCUSSION

Lipid metabolism in white adipose tissue played an essential role in maintaining energy homeostasis at high‐altitude area. In this study, WAT ER stress‐mediated lipolysis is enhanced in a rat model of high‐altitude hypoxia. Moreover, we found that increased FFA release results in liver lipid accumulation and liver dysfunction, which was attenuated by the inhibition of ER stress using 4‐PBA.

As ER membrane are located with a variety of lipid metabolism‐related enzymes and ER is the major site of lipid metabolism, ER is involved in the control of metabolic homeostasis via regulating lipid metabolism. Under normal conditions, ER in the adipocyte functions to meet the demands of protein synthesis and secretion, triglyceride synthesis, nascent lipid droplet formation, and nutrient sensing. However, ER function is overwhelmed and the UPR is activated under stressful conditions (Menikdiwela et al., 2019; Sikkeland et al., 2019). Therefore, perturbations in ER homeostasis exerts a vital pathogenic mechanism in multi metabolic disorders of adipose tissue (Khan & Wang, 2014; Suzuki et al., 2017). Adverse stimuli like hypoxia may pose challenges to adipocyte and induce ER stress. In the present study, continuous hypoxia exposure evoked ER stress in adipose tissue, evidenced by increased GRP78, CHOP, p‐PERK, and p‐IRE1α expression in rat WAT. Our finding is in accordance with previous studies showing that hypoxia exposure induce ER stress in 3 T3‐F442A and 3 T3‐L1 adipocytes (Mihai & Schroder, 2015). UPR pathways were activated to ameliorate the overload of unfolded proteins under ER stress, which in turn influence lipid metabolism (Song et al., 2016). The activation of ER stress in adipose tissue may further induce lipolysis and elevated circulating FFAs (Song et al., 2017).

To confirm the potential role of the ER stress and UPR in the modulation of the lipolysis, we treated rat with 4‐PBA, an ER stress inhibitor. 4‐PBA treatment led to significant reduction in lipolysis, which blocked the phosphorylation of HSL and perilipin. As the results from upstream regulation, 4‐PBA treatment then effectively reduced glycerol and FFA release from adipose tissue, suggesting that ER stress‐mediated lipolysis mainly by regulating cAMP‐PKA/HSL under hypoxia. Similar to our study, enhanced lipolysis and ER stress occurred in the visceral WAT and inhibition of ER stress alleviated lipolysis in a rat model of chronic kidney disease(Zhu et al., 2014). In addition, curcumin was reported to suppress the ER stress‐mediated lipolysis via cAMP/PKA/HSL pathway (Wang et al., 2016). Deng et al., also reported that ER stress involved lipolysis through up‐regulation of GRP78 and activation of phosphorylation status of PERK and eIF2α in rat adipocytes (Deng et al., 2012).

**FIGURE 6:**
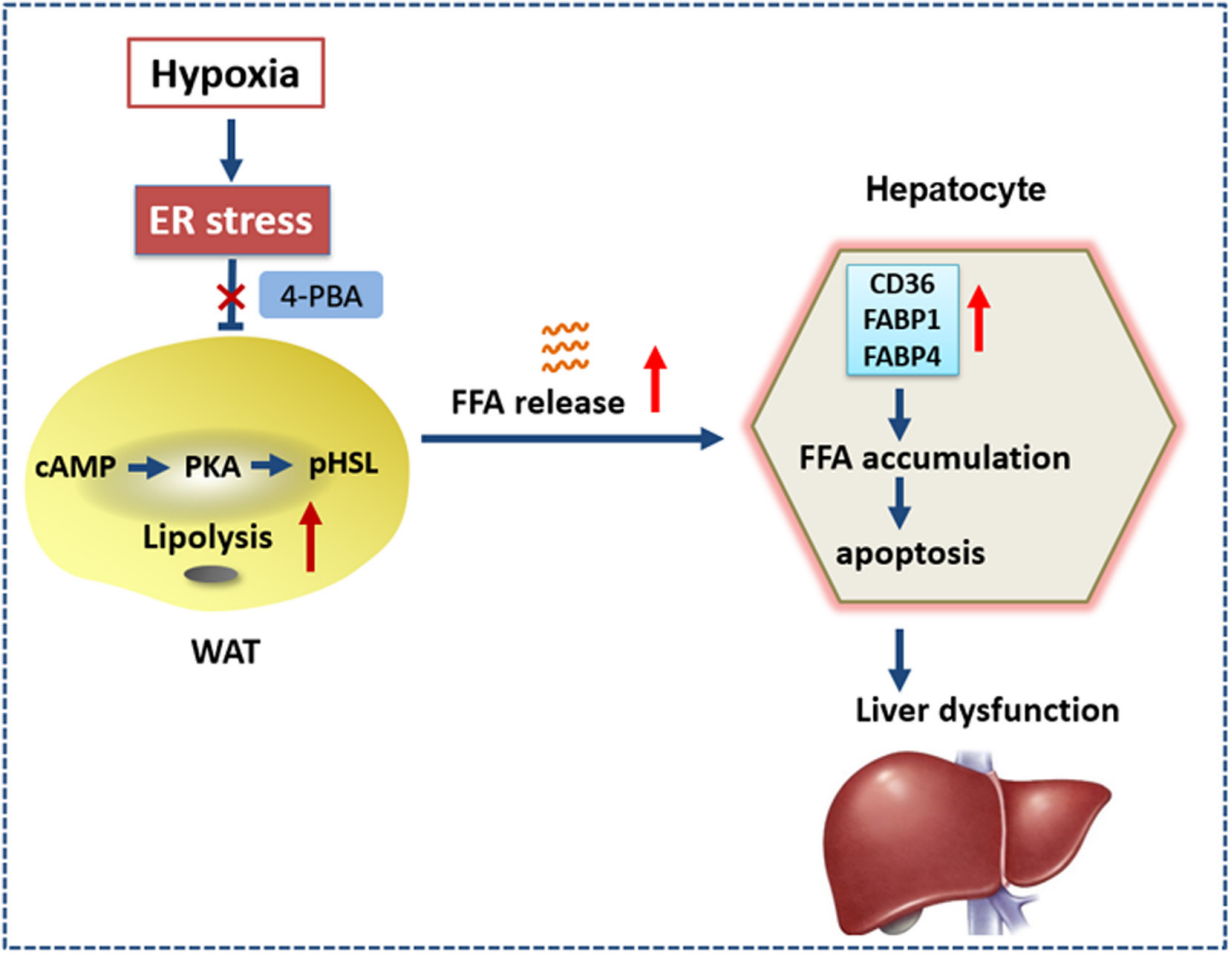
Protective mechanisms of 4‐PBA inhibit hypoxia‐induced lipolysis in WAT and lipid accumulation in the liver through regulating ER stress. Treatment with 4‐PBA, inhibitor of ER stress, effectively attenuated hypoxia‐induced lipolysis via cAMP‐PKA‐HSL/perilipin pathway. In addition, 4‐PBA treatment significantly attenuated liver injury and apoptosis, which is likely resulting from decreased liver lipid accumulation via inhibiting FFA transport.

Since the liver is the largest metabolic organ and regulates various physiological and metabolic processes, it also performs a key role in high‐altitude adaptation (Xu et al., 2019). Adipose dysfunction is closely associated with metabolism‐related liver diseases, an understanding of the interplay between tissues and these proposed mechanisms is still necessary (Da Silva Rosa et al., 2020). Accumulating data are pointing out the pathophysiological role of ectopic fat accumulation in different organs, including the liver (Bosy‐Westphal et al., 2019). In this study, the increased uptake of circulating lipids induced by WAT lipolysis significantly stimulated hepatic expression of lipid uptake and transport proteins CD36 and FABP4, which resulted in excess fatty acid uptake and lipid over accumulation in the liver. As a result, hypoxia‐treated rats displayed increased liver enzymes and hepatic apoptosis. As shown in Figure 6, 4‐PBA effectively attenuated hypoxia‐induced lipolysis via cAMP‐PKA‐HSL/perilipin pathway. The protective effect of 4‐PBA on liver injury and apoptosis, is likely resulting from decreased liver lipid accumulation via inhibiting FFA transport. Lines of evidence proved that excess FFA may modify the biology and function of hepatocyte and play an essential role in the pathogenesis liver dysfunction (Pereira et al., 2021). A high serum level of saturated FFAs is associated with hepatocyte lipo‐apoptosis (Takahara et al., 2017). In line with our fundings, Hubel, E., et al. found that repetitive Amiodarone treatment led to ER stress and aggravated lipolysis in adipose tissue while inducing a lipotoxic hepatic lipid environment and hepatic injury (Hubel et al., 2021).

In conclusion, enhanced ER stress‐mediated WAT lipolysis was observed in a rat model of high‐altitude hypoxia, which contributes to hepatic dysfunction and apoptosis through excess release of FFA. Our findings highlight the vital role of 4‐PBA in WAT lipolysis and liver dysfunction via regulating ER stress, which may provide novel insights into systemic metabolic disturbances in high‐altitude area.

## CONFLICT OF INTEREST

The authors declare no conflict of interests.

## Data Availability

The data that support the findings of this study are available on request from the corresponding author.
